# Linguistic analysis of the autobiographical memories of individuals with major depressive disorder

**DOI:** 10.1371/journal.pone.0207814

**Published:** 2018-11-26

**Authors:** Philip Himmelstein, Scott Barb, Mark A. Finlayson, Kymberly D. Young

**Affiliations:** 1 Department of Psychiatry, University of Pittsburgh School of Medicine, Pittsburgh, PA, United States of America; 2 School of Computing and Information Sciences, Florida International University, Miami, FL, United States of America; University of St Andrews, UNITED KINGDOM

## Abstract

**Background:**

Major depressive disorder (MDD) is characterized by biases in memory, attention, and cognition. The present study utilized the Linguistic Inquiry and Word Count (LIWC) to examine the content of specific autobiographical memories (AMs) recalled by individuals with MDD during an autobiographical memory task.

**Methods:**

We examined various features of the text (including use of affective, cognitive, and self-referential terms), as well as their associations with clinical and cognitive features of MDD (depression severity, autobiographical memory specificity, amygdala activity), in 45 unmedicated adults with MDD compared to 61 healthy controls.

**Results:**

When recalling positive memories MDD individuals used the word “I” less, fewer positive words, more words indicating present focus (present tense verbs), and fewer words overall to describe memories compared to controls. When recalling negative memories, MDD individuals used “I” more, more words indicating present focus, and more words overall to describe memories relative to controls. Depression severity was correlated with word count, the use of “I”, and words indicating present focus in negative memories and inversely correlated with word count and the use of “I” in positive memories. Autobiographical memory specificity was correlated with word count, the use of “I”, and words indicating present focus for positive memories and inversely correlated with the use of “I” and words indicating present focus for negative memories.

**Limitations:**

Due to the nature of AM recall, we could not control for the number of memories which participants recalled in each mnemonic category.

**Conclusions:**

Results align with literature implicating rumination and intensive self-focus in depression and suggest that interventions targeting specific word use may be therapeutically beneficial in the treatment of MDD.

## Introduction

Major depressive disorder (MDD) is a debilitating psychiatric illness affecting approximately 15% of individuals within a lifetime [1]. It is characterized by changes in affect, difficulties in social and cognitive functioning, and an increased risk for suicidal and self-harm behaviors [2, 3]. Current research indicates that impairments in MDD are cognitive in nature [4–10].

One method of examining cognition is through the study of language and memory. Previous research has largely focused on language and memory in MDD individually [5, 11–14], with only limited research looking at memory and language simultaneously within depressed individuals [15, 16]. One method of examining the connection between language and memory is by measuring or manipulating the affective valence of the recalled memory and examining whether linguistic structure changes as a result. While this research has been performed in healthy, stressed, and traumatized individuals [17–19], it has received limited attention in the context of depressive symptomatology and has not been performed in individuals with MDD [16]. The present study explores subcategories of language with the goals of 1) replicating previous findings of linguistic abnormalities in depression and 2) determining whether linguistic biases differ by memory valence.

It is a well-established and replicated finding that patients with MDD have impairments in autobiographical memory (AM) recall [20]. An AM is an episodic memory of an event that occurred to an individual at a specific place and time. AM can be further divided into specific and categorical memories [20]. A specific AM is a memory of a specific time or place (e.g., “I failed the test last Tuesday”), whereas a categorical AM summarizes a category of events without referencing a single example (e.g., “I have failed a lot of tests”). Research in this domain has shown that in addition to an overgenerality bias (consisting of more categorical and fewer specific AMs relative to controls; [20]), individuals with MDD also recall fewer positive memories and more negative memories [20–23]. Because of the importance of specific memory recall in daily life functioning [24–26], the present study focuses on the linguistic structure of specific memories in MDD and healthy individuals. While it has been established that the number of negative and positive memories recalled differs between healthy and depressed individuals [21], it remains an open question whether the content of these memories differs as well. Linguistic analysis provides a unique way to measure the content of memory to answer this question.

In the linguistic domain, research has found that impairments in the linguistic structure of depressed individuals can measure attention, emotion, cognitive processing, and social processes [15, 27–30], all of which show dysfunction in depression [9, 31]. One method of measuring linguistic structure is through the Linguistic Inquiry and Word Count (LIWC) program. The LIWC is a text analysis program that counts words that have been pre-categorized into semantic and syntactic categories [32, 33]. For example, one category includes all words that indicate an expression of negative emotion, including words suggesting “anxiety, anger, or sadness.”

Studies using LIWC have examined a number of linguistic categories within individuals with MDD including negation [15], pronouns [34], social activity and perception [13, 35], and emotion [17]. Because the number of linguistic categories that LIWC can examine is vast and our goal is to examine whether the linguistic contents of AM differs by memory valance, we choose to limit our main hypotheses to the three most enduring linguistic abnormalities found in depressed individuals. Specifically, we chose self-referential first person singular pronoun use (“I, me, my”) because it is perhaps the most well researched LIWC category in patients with depression yet has not been examined in relation to emotional memory valence [28], and we chose the frequency of negative and positive emotional word use to examine whether emotional biases exist in how depressed individuals describe positive, negative, and neutral valenced memories. All other analyses are exploratory. We also investigate the relationship between linguistic categories and symptom severity, autobiographical overgenerality, and amygdala activity. Amygdala activity was selected as it has been previously found to mediate the relationship between autobiographical overgenerality and depressive symptoms and may be a causal mechanism underlying recovery from MDD [23].

Higher usage of first person singular pronouns has been found in individuals with MDD relative to healthy controls during verbal and written memory recall and during everyday conversation [11, 12, 15, 16, 28, 36–38]. Furthermore, the use of first person singular pronouns is associated with increased self-focus, anxiety, neuroticism, and negative emotions in healthy individuals [11, 28, 37–42], suggesting excessive self-focus may contribute to depressive symptoms. However, to our knowledge no study has examined differences in first person singular pronoun usage based on affective memory valence in any population.

In healthy and depressed individuals, research has linked the use of negative and positive emotional words to the experience of negative or positive emotion [15, 18, 43–45], with depressed individuals using more negative emotion words and fewer positive emotion words than healthy individuals when writing in online depression communities, on Twitter, and when writing essays about personal experiences [12, 15,16, 36, 38, 45].

Several studies have used LIWC to examine emotionally valenced AMs in healthy individuals, and have confirmed that negative words are used more often to describe sad or stressful experiences and positive words are used more often to describe positive experiences [17–19]. Only one study to date has examined differences in positive and negative emotional word use by affective valence in depression [15]. Researchers classified over 400 posts in an online depression forum as either positive or negative in valence and found positively valenced posts had more positive emotion words, while negatively valenced posts had more negative emotion words. However, this study did not contain healthy controls and instead only compared use of affective words in depressed individuals. Hence, the present study aims to provide the missing link between depression, linguistic expression, and memories that differ by affective valence in comparison to healthy controls.

This is a secondary analysis on data from a study using functional magnetic resonance imaging (fMRI) to examine brain activity during emotionally valenced AM recall in patients with MDD [23]. We predict that individuals with depression will use more first person singular pronouns during negatively valenced memories. We also predict that individuals with depression will use more negative affective words and less positive affective words in all valenced memories compared to controls.

## Methods

Approval for this study was provided by the Western Institutional Review Board, which is the IRB of record for the Laureate Institute for Brain Research, where the study was conducted.

### Participants

One hundred and six medically healthy, right-handed individuals ages 18–55 entered one of two participant groups: psychiatrically healthy participants with no first degree relatives with a history of a mood disorder (n = 61; 31F, 30M), and unmedicated adults with MDD in a current major depressive episode according to the Diagnostic and Statistical Manual of Mental Disorders (DSM-IV-TR; n = 45; 25F, 20M). Volunteers, recruited from the community via advertisements, underwent medical and psychiatric screening evaluations at the Laureate Institute for Brain Research, which included the Structural Clinical Interview for *DSM-IV* disorders [46], and an unstructured diagnostic interview with a psychiatrist.

Exclusion criteria included current pregnancy, general fMRI exclusions, serious suicidal ideation, psychosis, major medical or neurological disorders, exposure to medication likely to influence cerebral function or blood flow within three weeks, and meeting DSM-IV-TR criteria for drug/alcohol abuse within the previous one year or for dependence (except nicotine) within the lifetime. Additional exclusion criteria applied to healthy control (HC) participants were current or past personal or family history of any major psychiatric disorder, or personal history of prescription psychotropic medication. After receiving a complete explanation of the study procedures, all participants provided written informed consent as approved by the Western Institutional Review Board (IRB). Participants received financial compensation for their participation.

### Autobiographical memory task

The data presented here were collected while participants were undergoing fMRI. Brain imaging results have been published elsewhere [23]. Prior to scanning participants were instructed as to the different types of AMs they might retrieve (according to conventional definitions used in the AM literature [20, 22, 47]) and informed their goal was to recall a *specific* AM, defined as a memory for an event that occurred at an identified place and did not last longer than one day. A categorical memory referred to a category of events containing a number of episodes without reference to one specific event (e.g., all exams failed without reference to one particular exam). An extended memory referred to an extended period of time without reference to a specific event within the time frame (e.g. a week-long vacation). A semantic memory was defined as a statement of fact without an associated event (e.g., ‘I’ve never been dancing’). Participants were provided with these definitions and examples verbally prior to entering the scanner and immediately prior to the start of each fMRI run.

A computerized version of the AM task [48] was developed for use during fMRI. Participants were presented 60 words (20 positive[e.g., success], 20 negative[e.g., danger], and 20 neutral [e.g., journal]) [49] using E-Prime software. During fMRI, participants were presented with a cue word for 12s and instructed to recall a past experience. Following the cue, participants rated the retrieved memory on the specificity (specific, categorical, extended, semantic, repeat, no memory) and the valence (negative, somewhat negative, neutral, somewhat positive, positive, no memory). Because the task was fixed so that participants had to respond to every question, “No Memory” was an option for the valence rating so that participants could indicate that they did not have a memory for a particular cue word, but still had the same timing during the task for fMRI analysis. Participants had 10s to assign each rating by scrolling to the relevant option and making their selection using a scroll wheel.

The AM recall condition was compared to a semantic example generation condition to control for abstract/general knowledge retrieval [50]. Participants were presented with an example generation cue word for 12s and instructed to think of at least seven examples from the presented category. Ten positive (e.g., flowers), 10 negative (e.g., villains), and 10 neutral categories (e.g., instruments) selected from the same word pool as the memory cue words) were presented. Following an example generation cue word, participants rated the ease with which they were able to generate examples (very easy, easy, somewhat easy, somewhat difficult, difficult, very difficult) and the number of examples they generated (0, 1–2, 3–4, 5–6, 7, 8 or more). Participants had 10s to select each rating. Memory and example generation cue words were selected from Bradley and Lang’s Affective Norms for English Words (ANEW; [51]. Words were matched across the affectively valenced word lists for frequency of use in spoken English (according to the standardized ratings provided in the ANEW). Following the presentation of each cue and each set of ratings, participants engaged in a riser detection task as a control for visual input/attention.

The order of memory and example cue word presentations was pseudo-randomized with restrictions on order presentation to prevent sequential presentations of a particular valence. Within each of ten runs, participants were presented with six memory cue words, three example generation cue words, and 18 riser letter strings in the order: cue word–riser–ratings–riser. Two computers time-linked to the image acquisition of the MRI scanner controlled stimulus presentation and behavioral response collection. Participants observed the stimuli using a mirror system attached to the head coil.

Following the scan, participants were presented with all AM cue words and asked to describe the memory for experimenter KY to corroborate participants’ specificity ratings. The experimenter was blind to diagnosis at the time of rating. In addition to standard memory categorizations of specific, categorical, extended, and semantic [20, 52], a memory was categorized as “can’t recall” if the participant was unable to recall the memory retrieved during fMRI. The experimenter typed all responses, verbatim, into a text document.

### LIWC

Typed responses were imported into an Excel 2016 spreadsheet where each individual response was associated with participant diagnosis, memory specificity, and rated valence. Responses that included no memory, or lacked a specificity or valence rating, were discarded. Spelling errors (which are attributable to the experimenter’s transcription) were corrected using Excel 2016’s built-in English spellcheck system. Because LIWC cannot take negations into consideration when coding for emotion words (i.e., I was not happy with my test results”), memories containing negations were removed from the analysis. Of the 5,253 memories recalled by all participants, only 12 contained negations (less than 1% of the data). The spreadsheet was then processed using LIWC2015 with segmentation on, generating, for each memory, counts for each of the 89 LIWC2015 output variables. Note that we did not include the 4 summary variables that are part of LIWC2015 because they are non-transparent dimensions and the specific composites are not available in the corresponding papers [33].

While we examined all possible LIWC variables, there were three main variables of interest that were central to our hypotheses. These were use of (1) personal pronouns, (2) positive emotion words, and (3) negative emotion words. We used the LIWC2015 internal English dictionary. The first person singular pronoun category included the words “I, me, and mine”. The positive emotion category includes 642 wordforms and patterns (including, e.g., “beautiful”, “favorite”, “respect”, and “roman*”), while the negative emotion category includes 746 wordforms and patterns (including, e.g., words such as “angry”, “jealous”, “sad”, and “unwelcom*”). Patterns that end with a star (‘*’) match any wordform that starts identically, i.e., the pattern “romanc*” matches both “romance” and “romances”.

### Data analysis

Data were analyzed using SPSS version 23. A repeated measures analysis of variance (ANOVA) was performed with the within-subjects factor of Cue Valence (positive, negative, neutral) and the between-subjects factor of Group (HC, MDD) for each of the LIWC variables for which the mean number was greater than one. Post hoc independent samples t-tests were performed when a significant Group x Valence interaction was found. The statistical criterion for significance was p>0.0011 (Bonferroni corrected for 45 comparisons). All memories categories (specific, categorical, extended, semantic) were combined and overall emotional memory recall, regardless of specificity, was examined.

We also correlated all LIWC variables with a) depression severity as measured by the Hamilton Depression Rating Scale (HDRS), b) AM specificity, and c) the amygdala response during positive and negative AM recall. The amygdala response was selected because we have implicated this response during positively valenced memory recall as a potential causal mechanism underlying onset and recovery from depression [23, 53]. Only correlations that survived correction for multiple comparisons (p<0.001) are reported.

## Results

We previously reported on the results of the specificity classifications, replicating previous findings that MDD participants had fewer specific AMs and more categorical AMs relative to controls, and this was particularly pronounced for positive memories [22].

The results from the repeated measures ANOVA can be found in Table 1. For variables that showed a significant Group x Valence interaction after correction for multiple comparisons, independent samples t-tests were performed. In addition to the three variables of interest, the variables Word Count, and Present Focus were also found to differ between the groups in the main analysis. With respect to the personal pronoun variable, the Group x Valence interaction did not survive correction for multiple comparisons (F(2,198) = 3.92, p = 0.02, η^2^_p_ = 0.04). However, there was a significant Group x Valence interaction (F(2,198) = 38.7, p<0.001; η^2^_p_ = 0.28) for use of the first person singular pronoun “I.”. MDD participants used the word “I” more than HCs when describing their negative memories (t(104) = 6.97, p<0.001, d = 1.41) and less when describing their positive memories (t(104) = 6.89, p<0.001, d = 1.39). The groups did not differ in the number of times “I” was used for neutral memories (t(99) = 2.10), p = 0.58, d = 0.42).

**Table 1:** Means and SD for linguistic variables for each memory valence and diagnosis.

	Memory Valence					
	Positive		Negative		Neutral	
Variable	HC	MDD	HC	MDD	HC	MDD
Use of first person singular pronouns	12.8 (2.78)	10.1 (3.57)	9.48 (5.27)	14.2 (3.97)	9.10 (4.42)	11.2 (5.19)
Use of the word "I"	12.3 (3.13)	8.46 (2.36)*	7.84 (4.57)	13.4 (3.20)*	7.43 (4.33)	9.36 (4.88)
Positive Emotional Words	4.48 (1.59)	2.26 (1.07)*	0.98 (0.94)	1.43 (1.33)	1.90 (2.12)	3.14 (5.15)
Negative Emotional words	1.08 (0.84)	1.04 (0.79)	6.40 (2.99)	5.86 (2.65)	4.15 (3.25)	2.12 (2.09)
Word Count	237 (78.0)	164 (55.0)*	86.4 (51.6)	166 (70.0)*	59.2 (35.7)	67.7 (39.8)
Present Focus	2.57 (1.62)	4.80 (2.46)*	3.01 (2.46)	5.19 (3.68)*	6.53 (4.29)	5.52 (3.66)

* = difference from the HC group at p ≤ .001

HC = healthy control; MDD = major depressive disorder

When examining the use positive and negative emotionally valenced words separately, there was a Valence x Group interaction for positive emotional words (F(2,198) = 14.2, p<0.001, η^2^_p_ = 0.13), while the interaction for negative emotional words did not survive corrections for multiple comparisons (F(2,198) = 4.59, p = 0.01, η^2^_p_ = 0.04). MDD participants used fewer positive words to describe their positive memories than HCs (t(104) = 8.09, p<0.001, d = 1.64), but did not differ in the number of positive words used to describe negative (t(104) = 2.01, p = 0.056, d = 0.39) or neutral memories (t(99) = 1.64, p = 0.14, d = 0.31).

Two additional variables showed a significant Group x Valence interaction that survived correction for multiple comparisons. Total word count differed between groups based on valence (F(2,198) = 46.9, p<0.001, η^2^_p_ = 0.32), with MDD participants describing their negative memories using more words (t(104) = 6.79, p<0.001, d = 1.29) and their positive memories using fewer words than HCs (t(104) = 5.33, p<0.001, d = 1.08). Groups did not differ in the number of words used to describe their neutral memories (t(99) = 1.13, p = 0.26, d = 0.22). Finally there was a significant Group x Valence interaction for the variable Present Focus (F(2,198) = 7.44, p = 0.001, η^2^_p_ = 0.07), which examines the use of verbs conveying the present tense. MDD participants had more of a present focus when recalling both positive (t(104) = 3.09, p = 0.003, d = 1.07) and negative (t(104) = 3.64, p<0.001, d = 0.70) memories relative to HCs, but did not differ in focus relative to HCs when recalling neutral memories (t(99) = 1.25, p = 0.21, d = 0.25).

**Fig 1:**
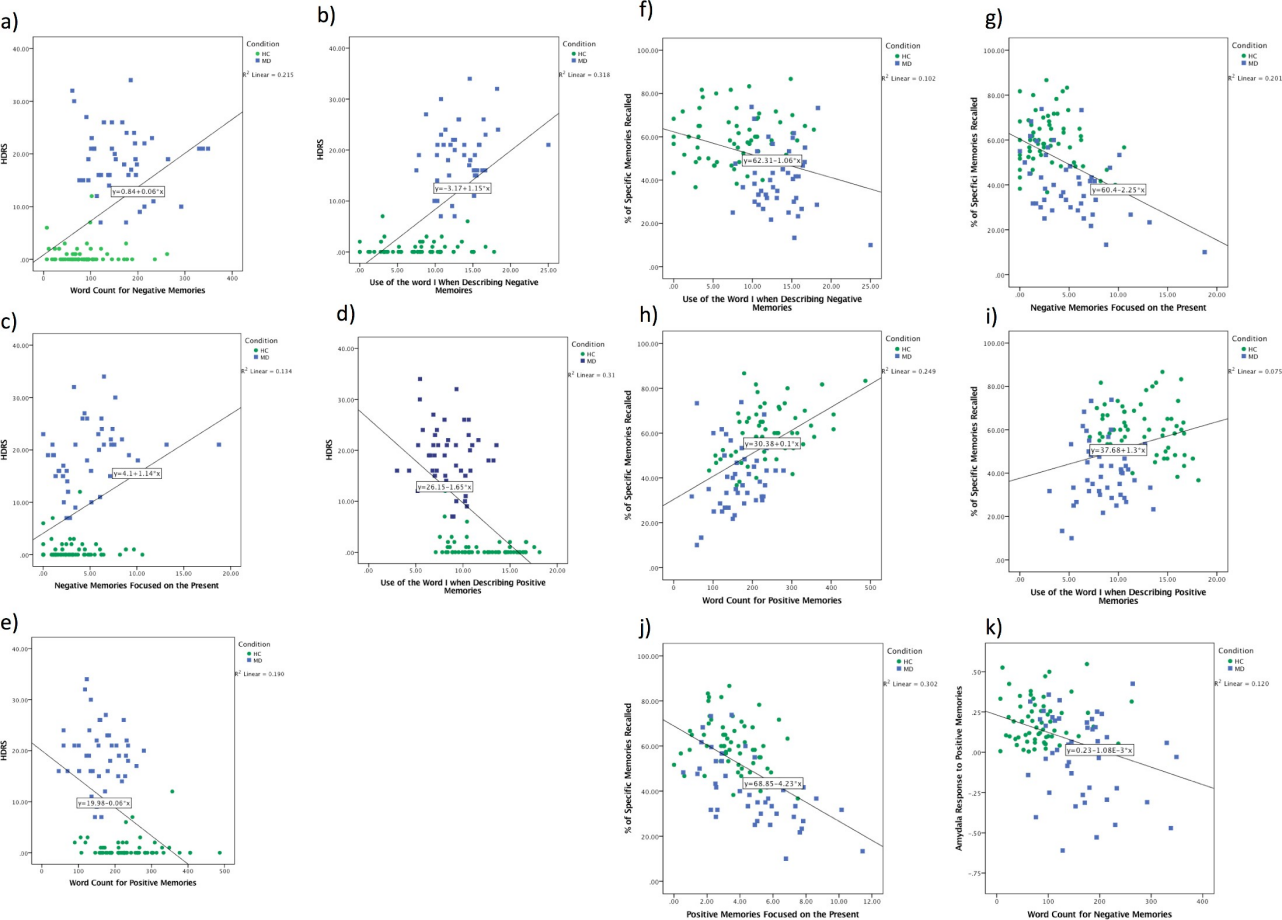
Correlation between linguistic and clinical/memory variables. (A-E) Correlation between depression severity as measured by the Hamilton Depression Ratings Scale (HDRS) and (A) word count for negative memories, B) use of “I” in negative memories, C) present focus for negative memories, D) use of “I” in positive memories and F) word count for positive memories. (F-J) Correlation between the percent of specific autobiographical memories recalled and (F) use of “I” in negative memories (G) present focus for negative memories, (H) word count for positive memories, (I) use of “I” in positive memories and (J) present focus for positive memories. (K) Correlation between the amygdala response to positive memories and word count for negative memories.

We also examined whether any of the LIWC variables were significantly correlated with a) depression severity as assessed by the HDRS, b) autobiographical overgenerality as assessed by the percent of specific memories recalled, and c) the amygdala response during both positive and negative specific memory recall. Only correlations that were significant at p<0.001 were considered significant after correction for multiple comparisons. As can be seen in Fig 1A–1E, depression severity was positively correlated with word count for negative memories, the use of I when describing negative memories, and the degree of present focus on negative memories. Depression severity was inversely correlated with word count for positive memories and the use of the word I when describing positive memories.

Fig 1F–1J shows the correlation between the LIWC variables and AM specificity. Specificity was inversely correlated with the use of I when describing negative memories and the degree of present focus on negative memories. Specificity was positively correlated with word count for positive memories, the use of the word I when describing positive memories, and the degree of present focus on positive memories.

Finally, amygdala activity during positive memory recall was negatively correlated with the word count to negative memories (Fig 1K). No other correlations survived correction for multiple comparisons.

## Discussion

In the current study, we found that in individuals with MDD relative to HCs the use of “I” was greater in negative memories, and that negative memories were longer, possibly suggesting that individuals with MDD display increased attention toward negative stimuli, a well repeated finding in the depression literature [54]. We found an inverse pattern involving positive memories, suggesting that individuals with MDD display decreased attention toward positive stimuli. The present findings are unique in that they underline the importance of accounting for memory valence when examining the words that individuals use. Depressed individuals used more present focused language when recalling positive and negative memories. Depression severity correlated positively with present focus in negative memories while AM specificity was inversely correlated with present focus in negative memories and positively correlated with present focus in positive memories. In the following paragraphs, we will examine each of these variables in greater detail and discuss their significance to the field of autobiographical memory and linguistic analysis.

### First person singular pronoun use

While the groups did not differ in their use of personal pronouns overall (“I, me, mine”), there was a significant group x valence interaction in the use of the word “I.” While depressed and healthy participants may not differ in the use of the personal pronouns “me and mine” they do significantly differ in their use of the personal pronoun “I,” supporting our hypothesis that use of some first person singular pronouns would differ between groups based on memory valence. Healthy individuals used “I” more when describing positive memories, while depressed individuals used “I’ more when describing negative memories.

Depressed individuals did not differ from healthy individuals in their use of “I” in when describing neutrally valenced memories. As previous research suggests that depressed individuals use “I” more as a result of ruminative self-focus [28], interpret neutral or ambiguous stimuli in a negatively valenced manor [9], and ruminative self-focus is involved in the maintenance of depression and negative thinking [54], we expected that MDD individuals might interpret neutral memories in a self-focused way and exhibit more use of “I” in neutral memories. This was not observed, possibly because of the disconnect between the neutral stimuli itself and the interpretation of the stimuli. Indeed, we have previously found that neutral cue words are more likely to cue recall of negatively valenced memories in depressed individuals while they are more likely to cue positive memories in healthy individuals [21], resulting in a low number of neutral memories in the present study (mean of 5 neutral memories recalled across groups).

Broadly, the finding that depressed individuals use “I” more often fits with a previous meta-analyses [28], and helps explain why the correlation found in this meta-analysis was relatively small (0.13), as memory valence likely acted as a confound in these studies, with depressed individuals recalling more negative memories that accounted for the observed correlation between depression and the use of “I”. More specifically, the observation that depressed individuals tend to use “I” more in negative memories fits with previous research that implicates the role of intensive self-focus in depression [55–57]. Depressed individuals also used “I” significantly less often in positively valenced memories compared to healthy individuals—a finding that aligns with research examining how depressed individuals perceive stimuli as salient. Previous work from our lab showed that depressed individuals experience increased activity in the salience network when recalling negative memories and decreased activity in this network when recalling positive memories compared to healthy controls [23]. Hence, it may be that depressed individuals perceive negative information as more salient and more relevant to the self and positive information as less salient and less relevant to the self. Further evidence of this relationship between self-focus and the use of the word “I” comes from research examining mood-congruent memory recall, which suggests that stimuli perceived as congruent with one’s current mood state are learned and remembered more often and more vividly than stimuli incongruent with one’s current mood state [58–60]. This effect has been shown to perpetuate the cycle of negative affect and negative memory recall in depressed individuals [61] and is particularly pronounced for information relating to the self [62, 63], suggesting that in our study, depressed individuals may be perceiving negative memories in a mood-congruent manner while discounting positive memories, and this effect may be enhanced for AMs relating to the self.

The present study also found a correlation between the use of the word “I’ and the percent of specific memories recalled. It is possible that ruminative self-focus may be a driving force behind both overgeneral memory and the use of the word “I” in the present study [64].

In addition, the present study found that depression severity positively correlated with the frequency of “I” in negative memories, while positive memories showed an inverse relationship. This is a key finding, as it aligns with an area of research suggesting that therapies in which depressed individuals decrease their use of the word “I” may be clinically useful in lowering depressive symptomology [65–69]. Findings in the present study indicate that it may be less therapeutically important to suggest that depressed individuals decrease their use of the word “I” generally and more beneficial for depressed individuals to reframe positive memories using “I” more often and reframe negative memories using “I” less often.

### Words indicating positive emotion

We also observed that when recalling positive memories, depressed individuals used significantly fewer positive words relative to controls. We did not observe this for neutral or negative memories. This finding aligns with previous literature suggesting that depressed individuals use fewer positive emotion words than healthy controls [12, 16, 17, 38] and aligns with theories that suggest depressed individuals have difficulty accessing mood-incongruent information and attending to positive stimuli [9, 23, 59, 60].

### Words indicating negative emotion

Contrary to our hypothesis, we did not find the frequency of negative emotional word use in positive, negative, or neutral memories to differ significantly between groups. Studies that have examined negative word use in relation to memories differing by affective valence in healthy individuals suggest that LIWC is able to detect differences in negative word use when memory valence is manipulated [16–19]. In regard to depression, previous research has largely observed higher negative emotion word usage in online depression forums and written narratives [12, 15, 36, 38], suggesting that that negative word usage in depressed individuals may be more characteristic of written rather than spoken language. Previous studies have hypothesized that because writing is slower than speaking, written language may allow time for response modulation that could affect the utterance of negative emotional word use [17]. In the current study, all memories were described orally to the experimenter, which may have decreased the utterance of negative words.

Importantly, the present study did not find correlations between AM specificity and positive or negative emotional word use, supporting previous research suggesting that the maintenance of overgeneral memory is related to thinking style and is unrelated to mood [64, 70].

### Word count

In the present study, we observed that total word count differed between groups based on valence, with depressed participants describing their negative memories using more words and their positive memories using fewer words than healthy individuals. These findings expand on previous mental health research [71, 72] and research comparing memories differing in affective valence in healthy individuals [19]. The observation that depressed individuals recalled negative memories that had more words than the negative memories of healthy individuals aligns with previous research suggesting that depressed individuals have a difficulty in disengaging with negative information [73–77]. We also found word count correlated with depression severity, such that word count increases with depression severity in negative memories, while word count decreases as depression severity increases in positive memories, further suggesting that differences in word count are related to the experience of depressive symptomatology.

In addition, we observed a relationship between the overgenerality effect and word count, such that individuals who recalled their positive specific memories with fewer words also tended to recall fewer specific memories. This may suggest that when depressed individuals do recall specific memories in response to a positive cue, the memories may receive less attention and therefore a shorter word count [21]. Another possibility is that depressed individuals may have poorer memory and may be less likely to recall mood incongruent information [14].

We also observed a relationship between amygdala activity and word count. Depressed individuals who tended to have decreased amygdala activity to positive memories also had a higher word count for negative memories relative to healthy controls. This is unsurprising, as both amygdala hypoactivity to positive memories and increased word count to negative memories are characteristic of depression [23]. A potential causal explanation for this observation involves research involving the default mode and salience networks, both of which are involved in the initiation and termination of repetitive thought in healthy and depressed individuals [78].

### Present focus

Finally, we observed that depressed individuals exhibited more words indicating present focus (present tense verbs) when recalling both positive and negative memories relative to healthy individuals. The observation that individuals with depression experience more present focus when recalling negative memories fits with the current literature on rumination—depressed individuals may be continually reliving their negative experiences such that negative experiences are felt more saliently, as if they are happening in the here and now [79,80, 54]. We also observed that as depression increased, so too did present focus in negative memories. This may reflect increasing levels of rumination in more severely depressed individuals, as depression severity has previously been shown to correlate with the frequency of ruminative thought [81].

The observation that MDD participants experience more present focus when recalling positive memories is intriguing. Given that blunted affect to positive stimuli is a hallmark of depression, one would expect that positive memories would be less focused on the present if they were felt less saliently [82]. The present findings suggest that present focus is elevated in depression, regardless of memory valence. Previous work suggests that two functionally distinct forms of self-focus exist: experiential self-focus, which is beneficial and aids in reinterpreting negative events, and analytical self-focus, which is associated with rumination, poor clinical course, and depression severity [64]. In our study, it is possible that depressed individuals are exhibiting analytical self-focus when recalling both negative and positive memories, which may be driving the use of present focus words. Future research is needed to fully explore this hypothesis. Furthermore, our findings align with research on the default mode network suggesting that this network is involved in initiating and suppressing thought, such that in our study depressed individuals may experience a decrease in cognitive control regardless of memory valence [81, 83].

### Limitations

One limitation of the present study is that memories were not verbalized during fMRI, but rather to the experimenter post-scan. This could lead to a situation in which the participant remembered their time in the scanner while they recalled the memory to the experimenter. We attempted to control for this by excluding any memories that participants either could not recall at post-scan or whose valence or specificity rating did not match that provided during imaging. Furthermore, due to the nature of AM retrieval, we could not control for the number of memories which participants recalled in each mnemonic category. Future studies may benefit from alternative cueing methods that elicit more balanced numbers of specifically targeted AM types. Finally, LIWC2015 is unable to account for negations when examining emotional words, such that “I did not feel happy” would be coded for the positive word “happy.” While the number of sentences containing these constructions in the current study was small, future studies should aim to recognize these complex patterns in order to fully understand the emotional tone used by participants.

### Future directions

Further research should compare categorical and specific memories, to determine whether the linguistic biases in memories observed in the present study are distinct from the biases that might be observed in specific or categorical memories. In addition, examining the linguistic structure of recalled memories that do not match the affective valence of a cue word (e.g., a participant is prompted with a positive cue word but recalls a negative memory), may also provide insights into the nature of depression. This technique may help to show why depressed individuals display deficits in encoding positive information and help determine how positive information is interpreted in a negatively valenced manor [9].

### Conclusion

The findings of the present study underline the cognitive nature of depression and implicate attention toward negative stimuli and away from positive stimuli as a key feature of depression present in and detectable across multiple categories of language. It is noteworthy that we observed that MDD participants used less positive emotion words to describe positive memories but did not use more negative emotion words to describe negative memories, suggesting that a ruminative, self-focused thinking style may be independent of negative emotional word use [54, 64]. More broadly, these results suggest that therapies that target specific facets of language may have therapeutic effects [34, 54].

## Supporting information

S1 Data File All memories included in the current analyses. (XLSX)
